# Robustly improved base editing efficiency of Cpf1 base editor using optimized cytidine deaminases

**DOI:** 10.1038/s41421-020-00195-5

**Published:** 2020-09-15

**Authors:** Siyu Chen, Yingqi Jia, Zhiquan Liu, Huanhuan Shan, Mao Chen, Hao Yu, Liangxue Lai, Zhanjun Li

**Affiliations:** 1 Key Laboratory of Zoonosis Research, Ministry of Education, College of Animal Science, Jilin University, 130062 Changchun, China; 2 CAS Key Laboratory of Regenerative Biology, Guangdong Provincial Key Laboratory of Stem Cell and Regenerative Medicine, South China Institute for Stem Cell Biology and Regenerative Medicine, Guangzhou Institutes of Biomedicine and Health, Chinese Academy of Sciences, 510530 Guangzhou, China; 3 Guangzhou Regenerative Medicine and Health Guang Dong Laboratory (GRMH-GDL), 510005 Guangzhou, China; 4 Institute for Stem Cell and Regeneration, Chinese Academy of Sciences, 100101 Beijing, China

**Keywords:** Molecular biology, DNA recombination

Dear Editor,

Programmable clustered regularly interspaced short palindromic repeats (CRISPR) associated Cpf1 endonucleases, also known as Cas12a, are single RNA-guided (crRNA) effectors^1^ that have been commonly utilized in various species to manipulate genome for their remarkable specificity^2–4^ and concise structures^1^. Cpf1 can recognize thymidine-rich (TTTV (V = A/G/C)) protospacer-adjacent motif (PAM) sequences and generates sticky breaks^5^, which enables it to be a complement to Cas9 in genome editing and broadens the genomic targeting scope. However, Cpf1-based base editors (BEs) generate lower editing efficiencies than SpCas9-based BE systems, due to the fact that the binding of Cpf1 nuclease to corresponding DNA targets is slack compared with that of in SpCas9^6^. In addition, previous studies have demonstrated the significantly improved gene knockout efficiency by modifications of crRNAs at the 3′ end of Cpf1, but it has not been systematically evaluated in Cpf1-based BEs^6–8^. Moreover, moderate base editing efficiency hinders Cpf1 from developing into generally employed BEs.

To date, Cpf1-associated BEs have been applied by a few teams in mammals revealing its undetectable editing efficiency at GC context in vivo^9^. In this study, we systematically assessed the veracity of three types of previously reported crRNA engineering (cr-HDV^8^, crRNA^tRNA^^7^, U-rich crRNA^6^) in HEK293T cells, while they failed to generate considerable editing efficiencies. Then, we reconstructed dLbCpf1-BE3 (dCpf1-BE3)^9^ with three distinctive deaminases (evoAPOBEC1, evoCDA1^10,11^, human APOBEC3A (A3A)^12–14^) to produce optimized dCpf1-based BEs. Here, we demonstrated that there is no significantly improved base editing frequency observed by using engineering of crRNAs, while the dramatically increased base editing efficiency was perceived by using cytidine deaminase optimized Cpf1 BE (dCpf1-eCDA1).

Firstly, the three crRNA configurations were constructed and tested at six genomic sites (Fig. 1a, Supplementary Fig. S1a). The results showed that U-rich crRNA slightly improved editing efficiency at all target sites ranging from 1.05- to 1.69-fold and significantly improved base editing efficiency at the *EMX1* site. The cr-HDV increased the base editing frequency up to 1.85-fold, while it reduced editing frequency at two sites (*CDKN2A* and *VEGFA-T*). The crRNA^tRNA^ failed to yield higher editing efficiency at most sites, except for *CDKN2A* (Fig. 1a, Supplementary Fig. S1b–g). Overall, there is no significantly improved base editing efficiencies observed by using modifications of crRNA in the dCpf1-BE3 system.

**Fig. 1:**
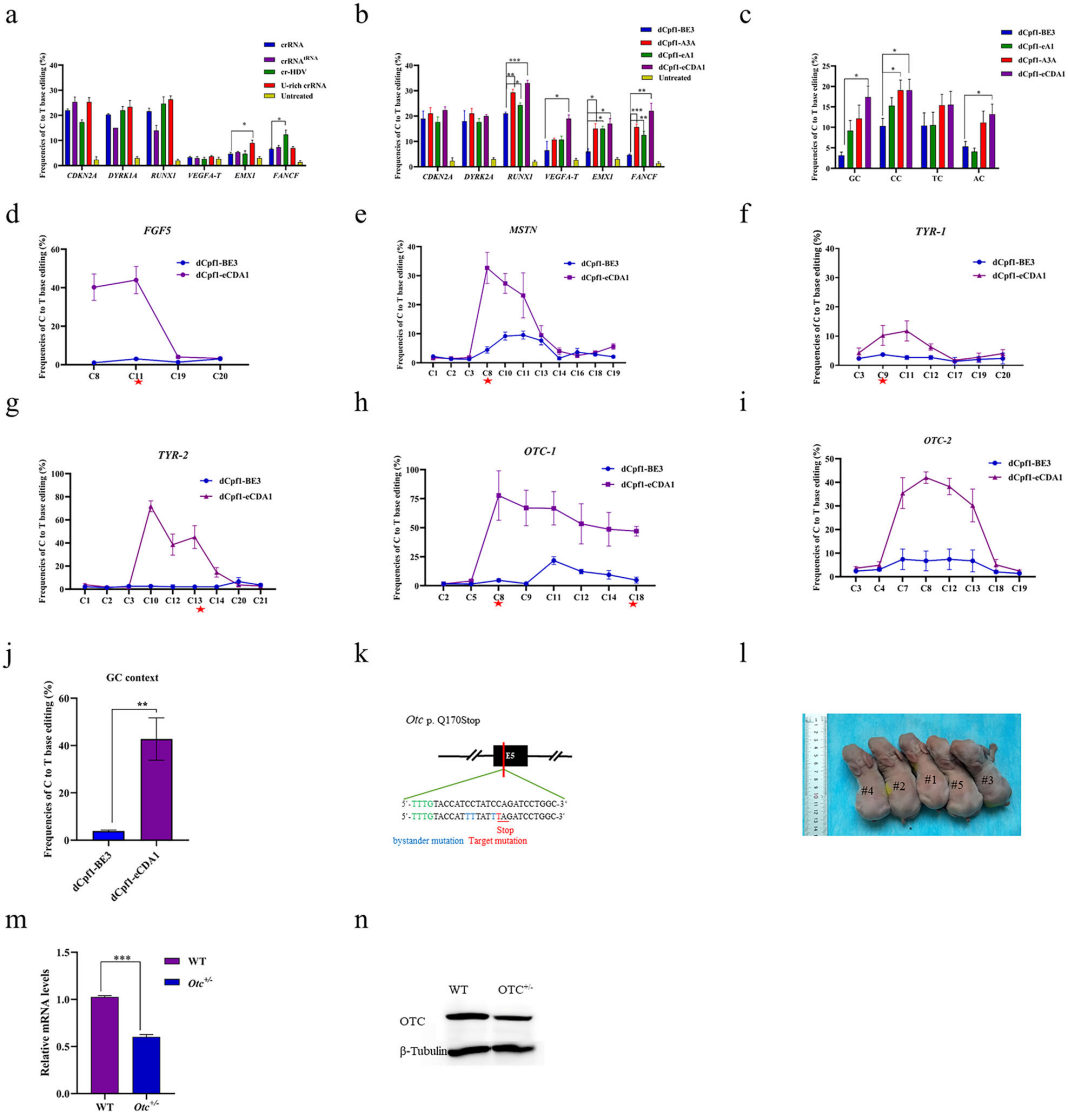
dCpf1-eCDA1 is applicable for highly efficient genome editing in vivo. **a** Comparison of base editing efficiencies of original crRNA, crRNA^tRNA^, U-rich-crRNA, and cr-HDV at six genomic sites in HEK293T cells. Editing efficiency was determined by analyzing Sanger sequencing chromatograms using EditR. The results are presented as mean value ± SEM of three independent experiments. **b** Comparison of dCpf1-BE3, dCpf1-A3A, dCpf1-eA1, and dCpf1-eCDA1 for base editing at six genomic sites in HEK293T cells. **c** The mean base editing efficiencies of dCpf1-BE3, dCpf1-A3A, dCpf1-eA1, and dCpf1-eCDA1 in GC, CC, TC, and AC contexts. **d**–**i** The base editing efficiency comparison of dCpf1-BE3 and dCpf1-eCDA1 at six genomic sites in rabbit embryos. The GC-context base editing efficiencies were marked with a red star. **j** The mean base editing efficiency comparison of dCpf1-BE3 and dCpf1-eCDA1 in GC context. **k** The target gRNA sequence of rabbit *Otc* locus in this study. The PAM and sgRNA target sequences are shown in green and black, respectively. Desired stop codon is underlined and marked in red. **l** The photos of five F0 rabbits generated by dCpf1-eCDA1. **m** The gene expression of *Otc*^+/−^ F0 rabbits was determined by RT-qPCR. **n** The protein level of *Otc* was determined by western blot. The anti-β-Tubulin antibody was used as the internal control. The data were analyzed with *t* tests using the GraphPad prism software 8.0. A probability of *P* < 0.05 was considered statistically significant. *P < 0.05, **P < 0.01, and ***P < 0.001.

Apart from the slack binding of Cpf1 nuclease to its corresponding DNA targets, the lower editing efficiency may derive from the moderate efficacy and context preference of rAPOBEC1 used in dCpf1-BE3. To validate this hypothesis, rAPOBEC1 in dCpf1-BE3 was replaced with more robust deaminases evoAPOBEC1, evoCDA1, and A3A to generate dLbCpf1-evoAPOBEC1, dLbCpf1-evoCDA1, and dLbCpf1-A3A BEs (hereafter termed as dCpf1-eA1, dCpf1-eCDA1, and dCpf1-A3A) (Supplementary Fig. S2a), respectively. The results showed that dCpf1-eCDA1 significantly improved editing efficiencies at four sites (*RUNX1*, *EMX1*, V*EGFA-T*, and *FANCF*). Both dCpf1-A3A and dCpf1-eA1 significantly enhanced editing efficiencies at three sites (*RUNX1*, *EMX1*, and *FANCF*) (Fig. 1b, Supplementary Figs. S2b–g and S3a–f). In detail, eCDA1 combined with Cpf1 significantly increased editing rates in all contexts except for TC context, similar to the results in Cas9^10^. dCpf1-A3A increased editing efficiency at all context, but only dramatically augmented efficiency in CC context, and dCpf1-eA1 did not enhance editing frequencies in different contexts (Fig. 1c). These results collectively demonstrated that dCpf1-eCDA1 and dCpf1-A3A performed point mutations more efficiently than did dCpf1-BE3 and dCpf1-eA1. In addition, analysis of editing window revealed the main editing window of dCpf1-BE3 ranges from positions 8 to 13, counting the base next to the PAM as position 1 (Supplementary Fig. S4a), consistent with that in previous report^9^. dCpf1-eA1 exhibits a similar editing window (positions 7–13) with dCpf1-BE3 (Supplementary Fig. S4b). The editing windows of dCpf1-A3A and dCpf1-eCDA1 are distinct from that of dCpf1-BE3. Maximal editing efficiency covers positions 6–20 for dCpf1-A3A (Supplementary Fig. S4c) and broadens to 5–21 for dCpf1-eCDA1 (Supplementary Fig. S4d). Due to its larger editing window, dCpf1-BE3 and dCpf1-eCDA1 could initiate base conversions more extensively, including the induction of stop codons and mutation of multiple sites within the gene regulatory regions.

Furthermore, top five potential off-targets (≤4 mismatches) for each genomic sites were predicted using online tool (http://www.rgenome.net/cas-offinder/) (Supplementary Table S1). Off-target editing events were detected for one crRNA at three sites. Strikingly, dCpf1-eCDA1 induced a lower level of base editing at predicted off-target sites compared with dCpf1-A3A and generated a similar or lower off-target editing level than dCpf1-eA1 (Supplementary Fig. S5a–f).

To further characterize the efficacy of dCpf1-induced BE in rabbits, we selected dCpf1-eCDA1 for its superior editing efficiencies and lower off-target efficiency relative to dCpf1-A3A in human cells. The mutagenesis frequencies were evaluated at six rabbit gene sites (Fig. 1d–i, Supplementary Table S1). The result showed a drastic increase in base editing efficiencies in dCpf1-eCDA1 compared with dCpf1-BE3 at all six sites and also in GC context as did in human cells (11.75–77.67% vs. 0–21.7%, respectively) (Fig. 1d–j, Supplementary Fig. S6a–f). Next, a crRNA targeting exon 5 of transcarbamylase (OTC) was designed to convert a C–G base pair into T–A to generate a premature stop codon (PTC) (Fig. 1k). Rabbit zygotes were injected with dCpf1-eCDA1 encoding messenger RNA and the corresponding crRNA, and five pups were generated in this study (Fig. 1l). The results showed the desired PTC mutation efficiencies from 3.7 to 61.3% (Supplementary Fig. S7a, b) were generated in those F0 rabbits, and gene expression of *Otc* in mutant offspring (#4) drastically decreased as determined by quantitative reverse transcription PCR and Western blot (Fig. 1m, n). In addition, there are no detectable off-target effects determined by Sanger sequencing in those F0 rabbit (Supplementary Fig. S7d, Table S1). Furthermore, the unwanted C to T conversions within the protospacer (bystander mutation)^15^ were detected in F0 rabbits (Supplementary Table S4, Fig. S7c). Even though bystander mutations could hinder precise editing toward target sites requiring high accuracy, they are innocuous in most cases. To solve the problem for accurate editing, we could choose target sites with only one C within editing window or further modify dCpf1-eCDA1 with mutations in evoCDA1 domain to narrow down its editing window^9,13,15^. Most importantly, there is a large suite of BEs available, each with different characteristics. For a given target sequence and application that has distinct needs, different BEs can be chosen to meet the specific requirement as recently reported^16^.

To our knowledge, this is the first animal model with high efficiency generated by dCpf1-eCDA1. In addition, there is no significant improvement in base editing efficiency by using modifications of crRNA, while robust improvement in base editing efficiency was observed by applying optimized cytidine deaminases to dCpf1-BE system.

## Supplementary information

Supplementary information

## Supplementary information

**Supplementary Information** accompanies the paper at (10.1038/s41421-020-00195-5).
