# Supplementary material for: Robustly improved base editing efficiency of Cpf1 base editor using optimized cytidine deaminases
Source: Cell Discov. 2020 Sep 15;6:62. doi: 10.1038/s41421-020-00195-5 (PMC7490413; doi:10.1038/s41421-020-00195-5)
Supplement: Supplementary file 1 — Supplementary information [file 41421_2020_195_MOESM1_ESM.pdf]

Materials and Methods

Ethics statement

New Zealand white rabbits were obtained from the Laboratory Animal Center of Jilin University (Changchun, China). All animal studies were conducted according to experimental practices and standards approved by the Animal Welfare and Research Ethics Committee at Jilin University.

Cell culture and DNA transfection

The human embryonic kidney (HEK) 293T cell line (Life Technologies) was cultured in Dulbecco's modified Eagle's medium (DMEM) supplemented with 10% fetal bovine serum (HyClone), 2 mM GlutaMAX (Life Technologies), 100 U/mL penicillin, and 100 mg/mL streptomycin, and was incubated at 37 °C with 5% CO_2. Cells were seeded into 6-well Poly-d-Lysine-coated plates (Corning). 12-15 h after plating, cells were transfected with 5 μL of Lipofectamine 3000 (ThermoFisher Scientific) using 1250 ng of base-editor plasmid, 1250 ng of guide RNA plasmid. Genomic DNA was extracted after 48h of transfection using the TIANamp Genomic DNA Kit (TIANGEN, Beijing, China) according to the manufacturer's instructions. DNA sequences including target base editing sites were amplified with primer pairs listed in Supplementary Table S2.

In vitro transcription

The dLbCpf1-BE3 (dCpf1-BE3) plasmid was a gift from Professor Jia Chen. The original crRNA was obtained from Addgene (#78957). dCpf1-eA1, dCpf1-A3A, dCpf1-eCDA1, cr-HDV, $crRNA^{tRNA}$ were commercially synthesized (Genscript, Beijing, China). The Cpf1 coding plasmids was linearized with *Not*I, and mRNA was synthesized using an *in vitro* RNA transcription kit (HiScribe™ T7 ARCA mRNA kit (with tailing), NEB). The crRNAs were *in vitro* transcripted using the MAXIscript T7 kit (Ambion) and purified using miRNeasy Mini Kit (Qiagen) according to the manufacturer’s instructions.

Microinjection of rabbit zygotes

The protocol for microinjection of pronuclear-stage zygotes has been described in detail in our published protocols[17]. Briefly, a mixture of dCpf1-eCDA1 and corresponding crRNA (200 ng/ul) and sgRNA (50 ng/ul) were microinjected into the cytoplasm of pronuclear-stage zygotes, and then transplanted into the surrogate mother.

Single embryo PCR amplification and rabbit genotyping

The injected embryos were collected at the blastocyst stage. Genomic DNA was extracted with an embryo lysis buffer (1% NP40) at 56 °C for 60 minutes and 95 °C for 10 minutes in a BIO-RAD PCR Amplifier, and then subjected to Sanger sequencing. Genomic DNA was extracted from ear clips of newborn rabbits for PCR genotyping and subjected to Sanger sequencing and target deep sequencing. All the primers are listed in Supplementary Table S2.

Targeted deep sequencing

Targeted sites were amplified from genomic DNA using Phusion polymerase (Thermo Fisher Scientific). The paired-end deep sequencing of PCR amplicons was performed by Sangon Biotech (Shanghai), using an Illumina MiSeq. Briefly, individual FASTQ files were analyzed commercially and each read was pairwise aligned to the crRNA sequence.

Real-time quantitative PCR (RT-qPCR)

Total RNA was isolated with TRNzol-A+ reagent (TIANGEN, Beijing, China) according to the manufacturer's instructions. cDNA was synthesized with DNAse I (Fermentas) treated total RNA using the BioRT cDNA First Stand Synthesis Kit (Bioer Technology, Hangzhou, China). Primers used for RT-qPCR are listed in Table S2. RT-qPCR was performed using the BioEasy SYBR Green I Real Time PCR Kit (Bioer Technology, Hangzhou, China) with the BIO-RAD IQ5 Multicolor Real-Time PCR Detection System. The relative gene expression normalized to the *Gapdh* was determined by $2^{-\Delta\Delta CT}$ formula. All the data of gene expression were performed three times, expressed as mean ± SEM.

Western Blotting

For Western blotting, liver tissues of WT and $Otc^{+/-}$ were homogenized in 500 μL of RIPA lysis buffer (Beyotime). The protein concentrations were measured by the Braford method (Bio-Rad). anti-*Otc* rabbit polyclonal antibody (1:400; abcam, catalog number ab55914) and anti-β Tubulin monoclonal antibody (1:4000; Proteintech, catalog number: 66240-1-Ig) were used as primary and internal control.

Statistical analysis

The base editing efficiencies of dCpf1-associated BEs were determined by using EditR (https://moriaritylab.shinyapps.io/editr_v10/?tdsourcetag=s_pcqq_aiomsg). All data are expressed as mean ± SEM, at least three individual determinations in all experiments. The data were

analyzed with t-tests using Graphpad prism software 8.0. A probability of $p<0.05$ was considered statistically significant. *$p<0.05$, **$p<0.01$, ***$p<0.001$， ****$p<0.0001$.

Funding

This study was financially supported by the National Key Research and Development Program of China Stem Cell and Translational Research (2019YFA0110702, 2017YFA0105101). The Program for Changjiang Scholars and Innovative Research Team in University (No.IRT_16R32). The Strategic Priority Research Program of the Chinese Academy of Sciences (XDA16030501, XDA16030503), Key Research & Development Program of Guangzhou Regenerative Medicine and Health Guangdong Laboratory (2018GZR110104004).

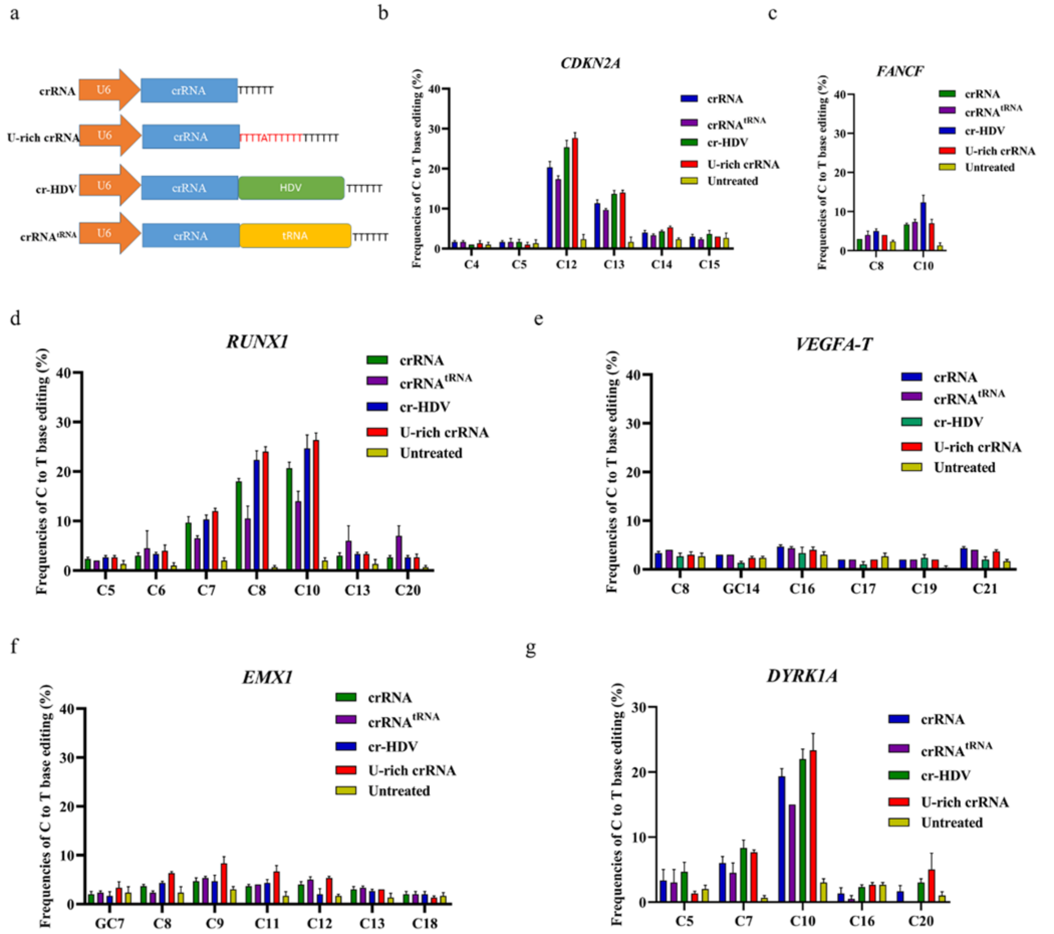

Supplementary Figure S1. Comparison of base editing efficiency of crRNA, crRNA^{tRNA}, cr-HDV and U-rich crRNA in HEK293T cells.

(a) Schematic representation of crRNA, crRNA^{tRNA}, cr-HDV and U-rich crRNA architectures.

(b-g) Base editing frequencies of (*CDKN2A*, *FANCF*, *RUNX*, *VEGFA-T*, *EMX1*, *DYRK1A*) were determined by using dCpf1-BE3 and crRNA, crRNA^{tRNA}, cr-HDV and U-rich crRNA systems.

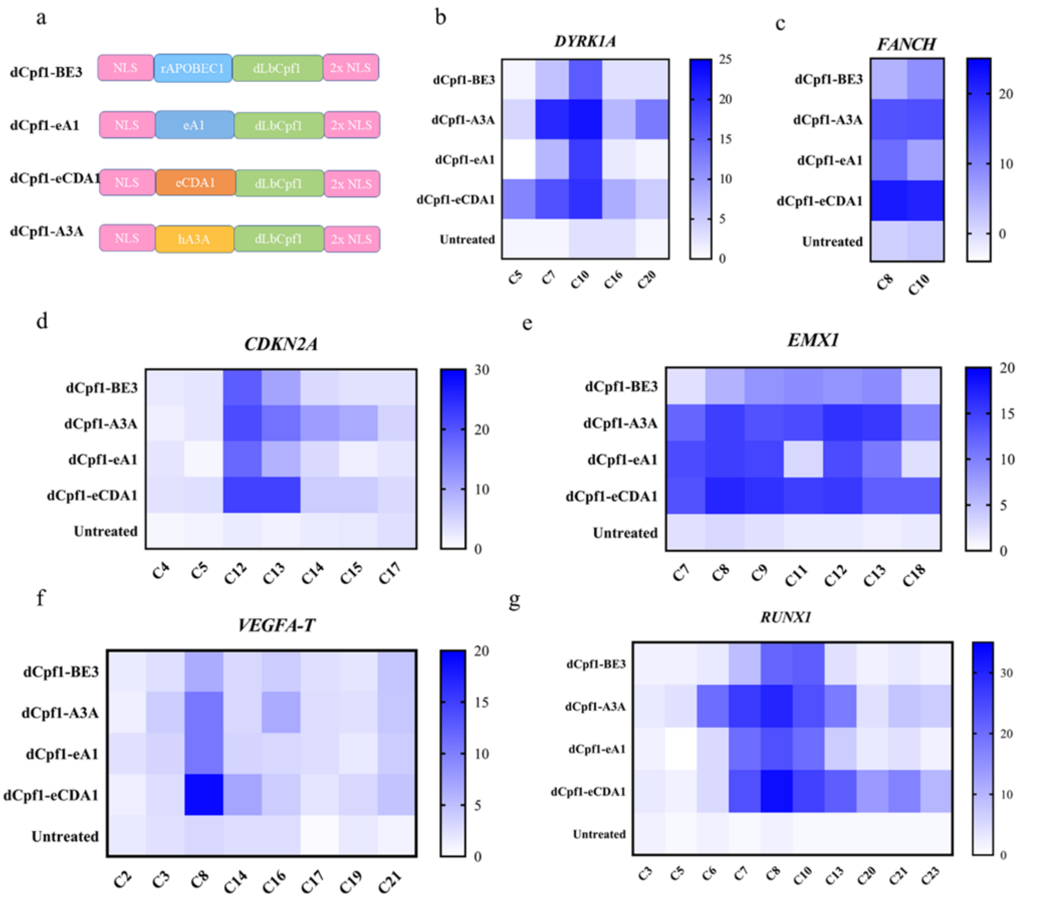

Supplementary Figure S2. Comparison of base editing efficiency of dCpf1-BE3, dCpf1-A3A, dCpf1-eA1 and dCpf1-eCDA1 in HEK293T cells.

(a) Schematic representation of dCpf1-BE3, dCpf1-A3A, dCpf1-eA1, dCpf1-eCDA1 architectures.

(b-g) Editing frequencies of the indicated bases were individually determined at different genomic target sites (*DYRK1A*, *FANCF*, *CDKN2A*, *EMX1*, *VEGFA-T*, *RUNX*) by using original crRNA and dCpf1-BE3, dCpf1-A3A, dCpf1-eA1 or dCpf1-eCDA1 systems.

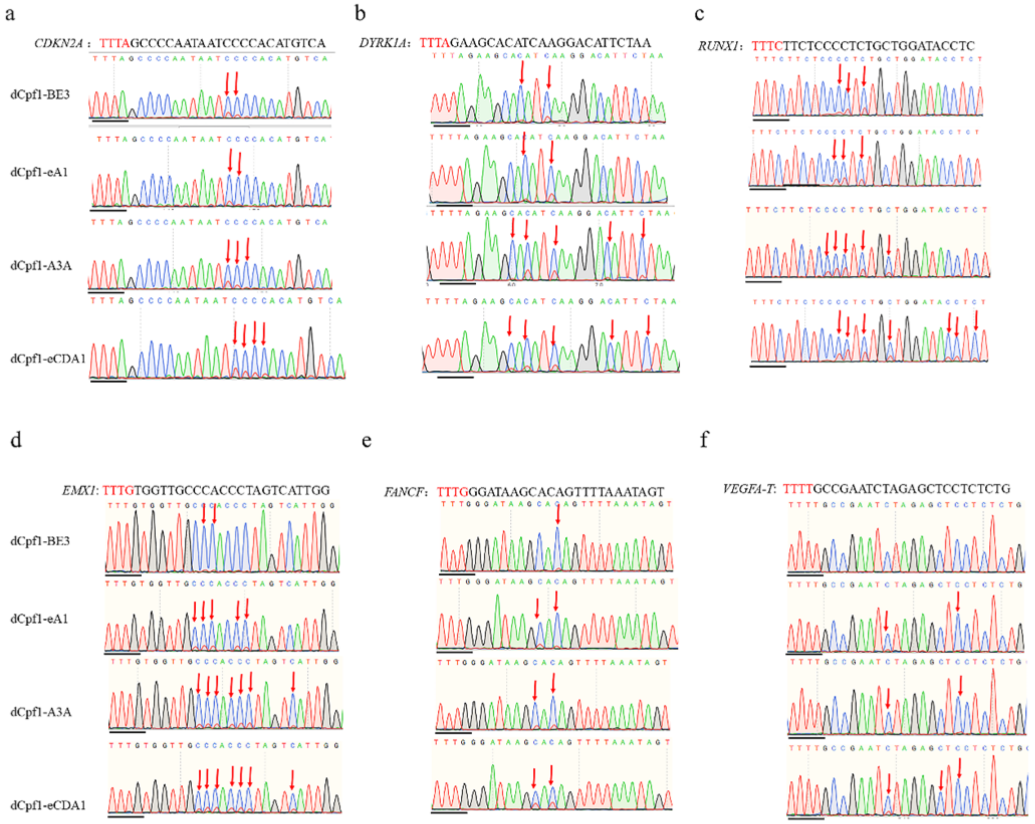

Supplementary Figure S3. Sanger sequencing chromatograms of six genomic targets in HEK293T cells.

(a-f) Presentative Sanger sequencing chromatograms of six genomic targets (*CDKN2A*, *DYRK1A*, *RUNX*, *VEGFA-T*, *FANCF*) by using original crRNA and dCpf1-BE3, dCpf1-A3A, dCpf1-eA1 and dCpf1-eCDA1 systems. PAM region was underlined and the mutated bases were marked with the red arrows.

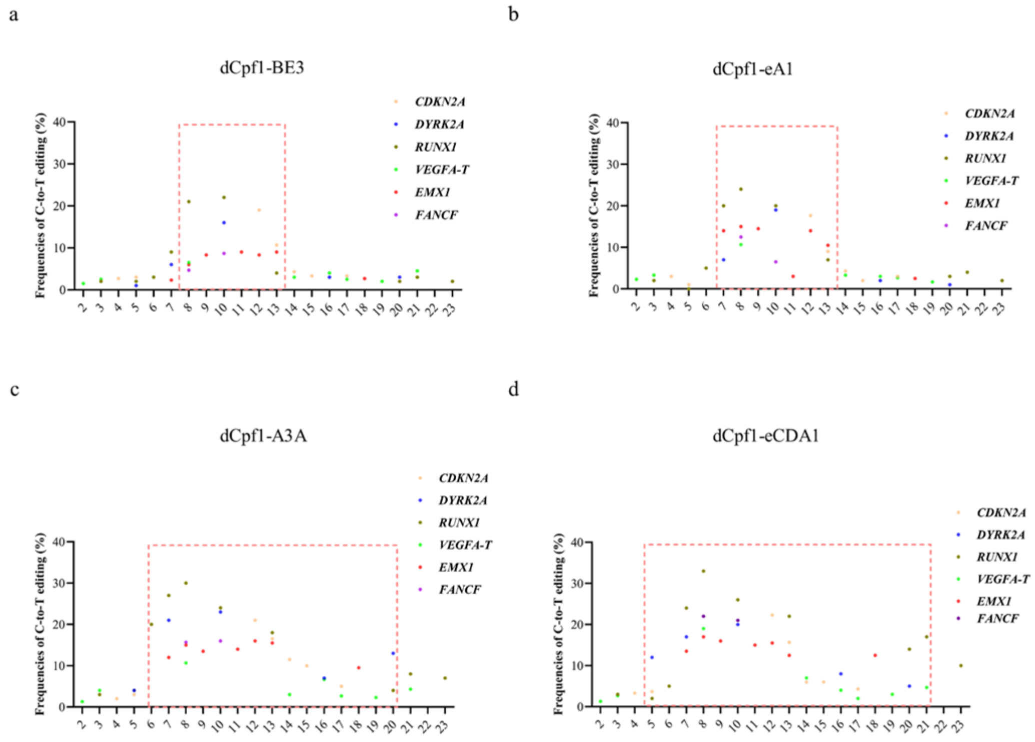

Supplementary Figure S4. Base editing activity window of dCpf1-BE3, dCpf1-A3A, dCpf1-eA1 and dCpf1-eCDA1 were determined in HEK293T cells.

(a) The base editing activity window of dCpf1-BE3 spans protospacer positions 8-13 (counting the first base proximal to PAM as 1).

(b) dCpf1-eA1 shares a similar 7-13 base editing window with dCpf1-BE3.

(c) The base editing activity window of dCpf1-A3A covers position 6-20.

(d) dCpf1-eCDA1 dramatically widens the editing window to position 5-21.

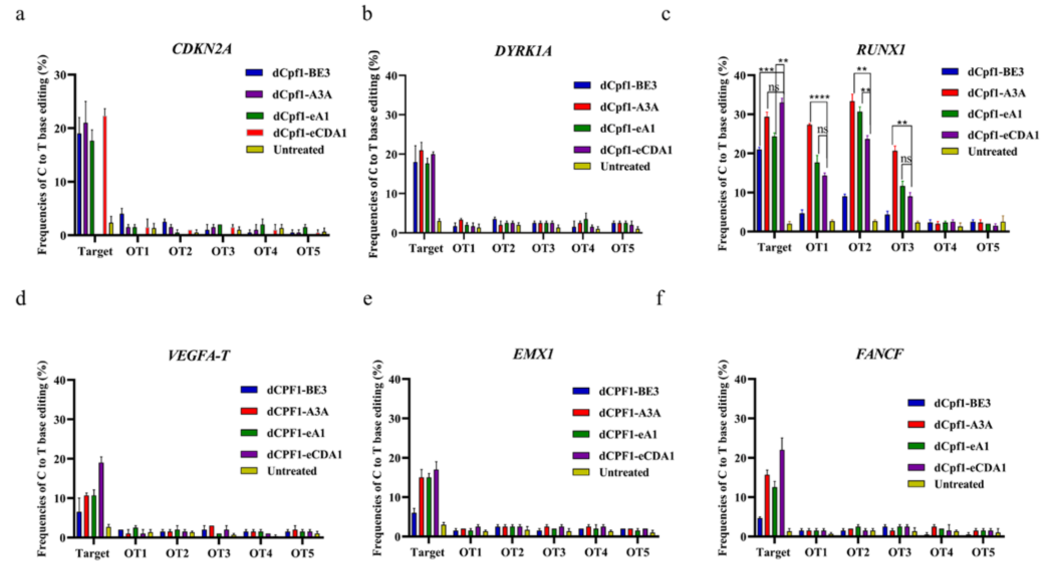

Supplementary Figure S5. The off-target analysis of dCpf1-BE3, dCpf1-A3A, dCpf1-eA1 and dCpf1-eCDA1 systems

(a-f) The C-to-T editing frequencies of the indicated cytosines generated by different base editors were determined at the indicated on- and off-target sites. The data were analyzed with t-tests using Graphpad prism software 8.0. A probability of $p<0.05$ was considered statistically significant. *p <0.05, **p <0.01, ***p <0.001.

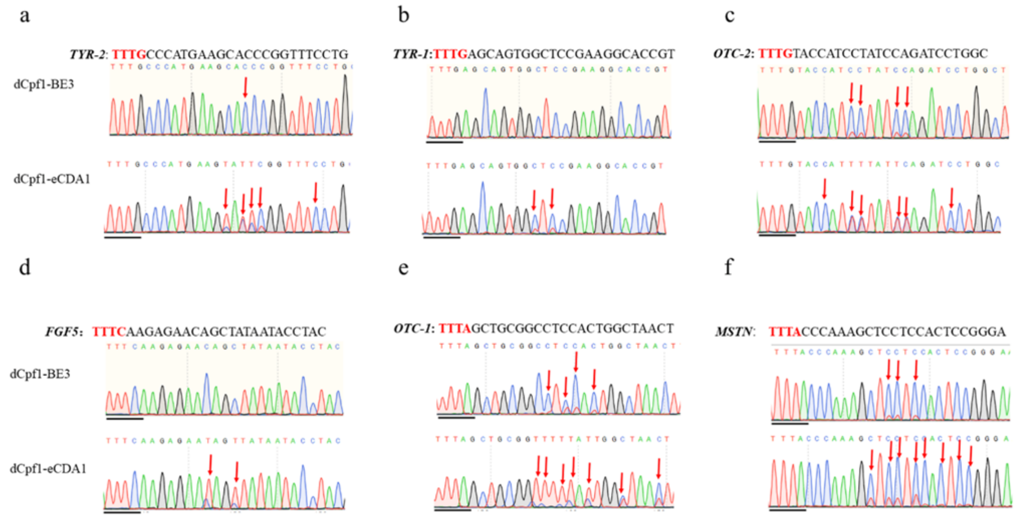

Supplementary Figure S6. Sanger sequencing chromatograms of six genomic targets in rabbit embryos by using dCpf1-BE3 and dCpf1-eCDA1.

(a-f) Presentative Sanger sequencing chromatograms of six genomic targets (*TYR-2*, *TYR-1*, *OTC-2*, *FGF5*, *OTC-1*, *MSTN*) by using dCpf1-BE3 or dCpf1-eCDA1. PAM region was underlined and the mutated bases were marked with the red arrows.

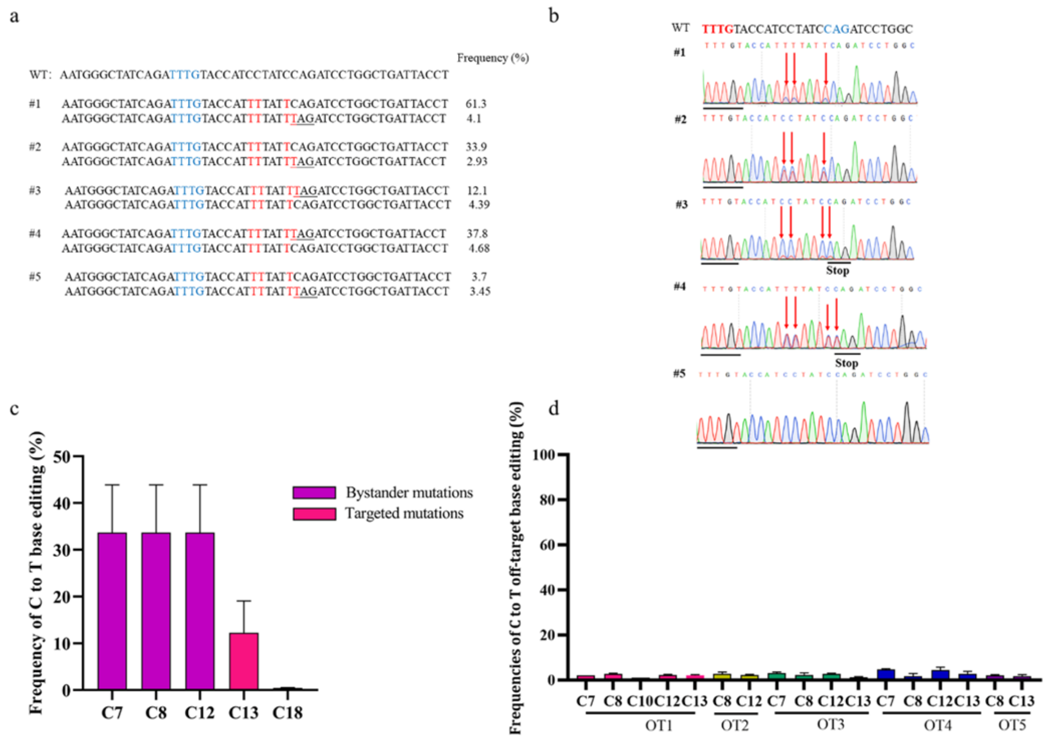

Supplementary Figure S7. The genotype and off-target of F0 rabbits

(a) Genotype of F0 rabbits were determined by deep sequencing.

(b) Presentative Sanger sequencing chromatograms of F0 rabbits.

(c) Analysis of bystander mutations and targeted mutations in F0 rabbits.

(d) The C-to-T editing frequencies of the indicated cytosines were individually determined at the indicated off-target sites in F0 rabbits.

Supplementary Tables

Supplementary Table S1 On- and off-target sites validated in HEK293T human cells and rabbit.

Name	CrRNA Sequences
CDKN2A	TTTAGCCCCAATAATCCCCACATGTCA
CDKN2A -OT1	TTTAGCgCCcATAATCCCCACgTGTCA
CDKN2A -OT2	TTTAatCCCtATAATCCCCACATGTCA
CDKN2A -OT3	TTTAatCCCtATAATCCCCACATGTCA
CDKN2A -OT4	TTTAGCCCCcATAATtCCCAtAgGTCA
CDKN2A -OT5	TTTA atCCCcATAgTCCCCACATGTCA
DYRK1A	TTTAGAAGCACATCAAGGACATTCTAA
DYRK1A -OT1	TTTT tAAaaACATCAAGGAtATTCTAA
DYRK1A -OT2	TTTG tcAGCAgATCAAGtACATTCTAA
DYRK1A -OT3	TTTAGgAGtACATCAAGGAatTTCTAA
DYRK1A -OT4	TTTTAAcCACATCAgGGACATgCTAA
DYRK1A -OT5	TTTTGAAGaAaATCAAGGACATTgTAT
RUNX1	TTTCTTCTCCCCTCTGCTGGATACCTC
RUNX1-OT1	TTTC TTCTCCCCTCTGCTGttTtCCTC
RUNX1-OT2	TTTC TTCTCCCCTCTGCTaGAagCCaC
RUNX1-OT3	TTTCTTCTCCCCTCTGaTGGAgACaaC
RUNX1-OT4	TTTGTTCTtCgCTCTGCTGttTACCTA
RUNX1-OT5	TTTA TTCTCCaCTCccCTGtATACCTC
VEGFA-T	TTTT GCCGAATCTAGAGCTCCTCTCTG
VEGFA-T-OT1	TTTGGggGAtTCcAGAGCTCCTCTCTG
VEGFA-T-OT2	TTTTGCCtAATCTAGcGgTCtTCTtTG
VEGFA-T-OT3	TTTTcaCaAATCTtGAGCTCCTCgCTG
VEGFA-T-OT4	TTTTtttGAATCTAcAaCTCCTCTCTG
VEGFA-T-OT5	TTTGGaCaAATCTAagGCTCCTCTtTG
EMX1	TTTGTGGTTGCCCACCCTAGTCATTGG
EMX1-OT1	TTTC TGGcTGCCCtCaCTAcTCATTaG
EMX1-OT2	TTTC TGGcTGggCTCCCTAGTCATcGG
EMX1-OT3	TTTG TtGTTGCtCACtgTAGTCATTGt
EMX1-OT4	TTTG TtcTTGaCaTCCCTAGTCATTGG
EMX1-OT5	TTTA TaGTTGCaCTcCCTAGggATTGG
FANCF	TTTGGGATAAGCACAGTTTTAAATAGT
FANCF -OT1	TTTG tGCTAAGCACtGTTTTAAATgGT
FANCF -OT2	TTTT GGAcAAGgACAGTaTTAAATAGC

FANCF -OT3	TTTT GacaAAGCACtaTTTTAAATAGT
FANCF -OT4	TTTA GGATAAGCAacaTTTTAAAaAtT
FANCF -OT5	TTTT GGATAAcCACAaTTTaAAAaAaT
OTC-1	TTTAGCTGCGGCCTCCACTGGCTAACT
OTC-2	TTTGTACCATCCTATCCAGATCCTGGC
MSTN	TTTACCCAAAGCTCCTCCACTCCGGGA
TYR-1	TTTGAGCAGTGGCTCCGAAGGCACCGT
TYR-2	TTTGCCCATGAAGCACCCGGTTTCCTG
FGF5	TTTCAAGAGAACAGCTATAATACCTAC
OTC-2-OT1	TTTGTAaCcTCCTcTCCAGcTCCTGGC
OTC-2-OT2	TTTATACCATaCTtgCttGATCCTGGC
OTC-2-OT3	TTTCTACCATCCTATCCtGtTtCTGaa
OTC-2-OT4	TTTTTACCATCCaATCCAttTCCTGta
OTC-2-OT5	TTTGTAgCATtCTAagCAtATCCTGGC

PAM was Marked in blue. Mismatches in off-target sites were presented in lowercase.

Supplementary Table S2 Primers used in this study to amplify genomic target sequences.

Target site	Primer sequence
CDKN2A-h-F	TAAACTGCTTGGGTGACAGG
CDKN2A-h-R	CAATCATGGCAGAAGGCAAAG
CDKN2A -OT1-F	AATGGCTTCACCAAAGAAAA
CDKN2A -OT1-R	ATGCTGTTATGAAGAAATACCC
CDKN2A -OT2-F	TCCAATGCCTGTATCCCCAT
CDKN2A -OT2-R	CGCTATGACGAAATATGCAA
CDKN2A -OT3-F	AGTAGTGGTTGTGGTTGCCA
CDKN2A -OT3-R	CTCACAGTTTCGCAAGGCTG
CDKN2A -OT4-F	GACAGCTATTTCTTATCTAGACTTC
CDKN2A -OT4-R	ATCTACGGTATTAGTCTGTTCT
CDKN2A -OT5-F	GCATTGAATCCATAGATTAC
CDKN2A -OT5-R	ATTAGTCCGTTTTCACACTG
DYRK1A-h-F	ACCTTGTCACACACAATGAAAC
DYRK1A-h-R	CGTTATGCATTCACCATCTTCTG
DYRK1A -OT1-F	CTTCTAAATTTGACTGGAAG
DYRK1A -OT1-R	GGTCAGTGTTCTGAAAATTA
DYRK1A -OT2-F	TTCTGAATCAGTTTCCTCTC
DYRK1A -OT2-R	CAGAAAGCTAAAGCAGCACT
DYRK1A -OT3-F	AGAATACAATTCGTTTGCAC
DYRK1A -OT3-R	AGAATACAATTCGTTTGCAC
DYRK1A -OT4-F	GATTGAGATTAGCTCACTGA
DYRK1A -OT4-R	AACTTTGGGGAGAAATACAA

DYRK1A -OT5-F	GATCTTCTGTATTCTACCTTGA
DYRK1A -OT5-R	GGCTTATATGTAACTCTGCA
RUNX1-h-F-	CTTTGGGCCTCATAAACAAC
RUNX1-h-R-	GGTGAAACAAGCTGCCATTT
RUNX1-OT1-F	GAATTGTCCATTTACACTGC
RUNX1-OT1-R	ATTTCCCTGACATTCCAACA
RUNX1-OT2-F	CCATAACAAGTTGCATGTAA
RUNX1-OT2-R	GACACAAACAGCTTAACCCA
RUNX1-OT3-F	TCTGCCTTCAGCGAAGTCGC
RUNX1-OT3-R	CCTGGGAGCACTGTGCAGAGTA
RUNX1-OT4-F	CCTCCAGTTAGGAACCACTA
RUNX1-OT4-R	GCTGCAACTAACCTACTGAG
RUNX1-OT5-F	AGTAAGTTCCCAGAACAAGT
RUNX1-OT5-R	ATGATTGAGCAAAGAACACC
VEGFA-T-h-F	TGAGGGAGTAGAGGGTGTTAAG
VEGFA-T-h-R	GGGTTACTTCAGAGCAAAGGAG
VEGFA-T-OT1-F	CCACATCATCTCACATACCTCTT
VEGFA-T-OT1-R	TCAGCACTGAATCCCACATC
VEGFA-T-OT2-F	CTTGGAGAGCAGTACCACTTAC
VEGFA-T-OT2-R	GAGGGAAGGAGACACAGAATTG
VEGFA-T-OT3-F	CATCCTTCATCTCCTCACCTAC
VEGFA-T-OT3-R	GATGAGCCCACATTGCTTATG
VEGFA-T-OT4-F	CTTCCCTGATAACGCAACCT
VEGFA-T-OT4-R	CAGCTGCCTATCCATCCTTAC
VEGFA-T-OT5-F	CCAAGCACAAGTGGTCTGTATC
VEGFA-T-OT5-R	CCTGCTACCCTAATCTCTTCCT
EMX1-h-F	GGTAACTGTAGAAAGAGGGCTTTA
EMX1-h-R	TGCTTGTCCCTCTGTCAATG
EMX1-OT1-F	CCGATACCTGATTATTTTCA
EMX1-OT1-R	GAGTAGAGATGTTTCTTTCTGT
EMX1-OT2-F	TAATGAGAGATGCTAGGTTT
EMX1-OT2-R	GACAGGTTCCACAATAGGTT
EMX1-OT3-F	CAAGGCCGACTTTAGCTTTT
EMX1-OT3-R	AAGTGACTTTTCAGAGGCAG
EMX1-OT4-F	GATAAAGGGATAAGGAACCT
EMX1-OT4-R	CCACAGGAAACTAATACAGG
EMX1-OT5-F	ACTGGGGGACCTGTAGGCTA
EMX1-OT5-R	GCTTCCTTGCAGTCCTTGTT
FANCF-h-F	AATGAGACAGATGAGGTGGTTG
FANCF-h-R	CACATGGAAGGCCAAGAAGA
FANCF -OT1-F	AGACTTGCCAGATGTTCCAC
FANCF -OT1-R	AAGTATGTGGTCCACATTCA
FANCF -OT2-F	TGAGGATGCACAAAGAAACT

FANCF -OT2-R	TATCATGCTCCCCTATCTGG
FANCF -OT3-F	GCTCCTAATTTTAGGCAATA
FANCF -OT3-R	GAGAAATTTACTCAACATAGCC
FANCF -OT4-F	ATGTACTCTGCATCAGCTGA
FANCF -OT4-R	TAAGACACTGTTTCAGACACT
FANCF -OT5-F	GATACCACTACAGGGGCTCC
FANCF -OT5-R	CCCCTTCTGTTGCTTGCAAT
OTC-1-r-F	GATCCCTCACTGCAATCAGAA
OTC-1-r-R	CACTACAGTTCCCAAGACAGAC
OTC-2-r-F	GTTTGCCACAGAGTGTTGTC
OTC-2-r-R	GGCTTGTTCATCAGCATTCTC
MSTN-r-F	TGAGGCTGTGAAGGCATAAG
MSTN-r-R	GGAGCAAGAGCCAATCATAGA
TYR-1-r-F	AGCATTTGCCCAGGTCTT
TYR-1-r-R	GGTAACTGTAGAAAGAGGGCTTTA
TYR-2-r-F	GCGACTCTTGGTGAGGAAA
TYR-2-r-R	AAAGATGCTGGGCTGAGTAG
FGF5-r-F	CGACTGCTTGAATCTTGGTAGA
FGF5-r-R	CCAAGCACCTAGTGAACAGAA
OTC-2-OT1-F	AGGGTCAATTCCTTTCCTTCC
OTC-2-OT1-R	CTCCTTCCCTCTCTTGCTTATTT
OTC-2-OT2-F	TGGGCACCAGTTCAAATCTC
OTC-2-OT2-R	CCTTGACCTGTTTCCTGCTTAT
OTC-2-OT3-F	CTGATGGTACAACTCCGTCATC
OTC-2-OT3-R	AAGCTACATGTTGGCACTAGAA
OTC-2-OT4-F	CAGAGGAGAGTAGGAGCATAGT
OTC-2-OT4-R	GGAGAGAGATGGAGACAGAGAT
OTC-2-OT5-F	CATATTCCTACACTGCCTCTCTTG
OTC-2-OT5-R	CCCTTACTGCTACATTCCTGTG
OTC-2-qPCR-F	GCTAACTTGCTGTGGAGTCTT
OTC-2-qPCR-R	GCATTTCTAAGAGCTGTGTTGTTC
GAPDH- qPCR-F	ATCCATTCATTGACCTCCACTAC
GAPDH- qPCR-R	GTACTGGGCACCAGCATCAC

Supplementary Table S3 Summary of embryonic development and base editors used.

Target sites	No. of zygotes	No. of 2-cell (%)[a]	No.of blastocysts (%)[a]	No. of mutants (%)[b]	Mean editing efficiency (%)[b]	Base editors
OTC-1	22	18(82)	12(55)	7(58)	21.7	dCpf1-BE3
	23	19(83)	10(53)	6(60)	77.67	dCpf1-eCDA1
OTC-2	20	17(85)	12(60)	3(25)	6.67	dCpf1-BE3
	20	18(90)	9(45)	6(67)	42	dCpf1-eCDA1
MSTN	22	18(82)	12(55)	9(75)	4.44	dCpf1-BE3
	27	24(89)	10(37)	6(60)	32.67	dCpf1-eCDA1
TYR-1	20	16(80)	12(60)	0(0)	0	dCpf1-BE3
	19	17(89)	11(58)	5(45)	11.75	dCpf1-eCDA1
TYR-2	19	16(84)	9(47)	4(44)	0	dCpf1-BE3
	22	19(86)	10(45)	7(70)	71.75	dCpf1-eCDA1
FGF5	20	17(85)	12(60)	0(0)	0	dCpf1-BE3
	20	14(70)	7(35)	7(100)	44	dCpf1-eCDA1

a. Calculated from No. of zygotes.

b. Calculated from No. of blastocysts.

Supplementary Table S4 Generation of founder rabbits using dCpf1-eCDA1.

Target Site	Mutant ratio(%)				
	No.of transplanted embryos	No.of offspring	No.of Mutants	No.of Bystander mutants	No.of PTC mutation (efficiency>10%)
OTC-2	25	5	5(100)	5(100)	2(40)
